# Validation of the Bluebelle Wound Healing Questionnaire for assessment of surgical‐site infection in closed primary wounds after hospital discharge

**DOI:** 10.1002/bjs.11008

**Published:** 2018-12-17

**Authors:** Bluebelle Study Group, Rhiannon Macefield, Jane Blazeby, Barnaby Reeves, Sara Brookes, Kerry Avery, Chris Rogers, Mark Woodward, Nicky Welton, Leila Rooshenas, Jonathan Mathers, Andrew Torrance, Anne Pullyblank, Robert Longman, Richard Lovegrove, Tim Draycott, Thomas Pinkney, Rachael Gooberman‐Hill, Jenny Donovan, Joanna Coast, Melanie Calvert, Natalie Blencowe, Lazaros Andronis, Dimitrios Siassakos, Caroline Pope, Madeleine Clout, Kate Ashton, Lucy Ellis, Christel McMullan, Rosie Harris, Daisy Elliott, Jo Dumville, Benjamin Waterhouse, Sean Strong, William Seligman, Lloyd Rickard, Samir Pathak, Anwar Owais, Jamie O'Callaghan, Stephen O'Brien, Dmitri Nepogodiev, Khaldoun Nadi, Charlotte Murkin, Tonia Munder, Tom Milne, David Messenger, Matthew Mason, Morwena Marshall, Jessica Lloyd, Jeffrey Lim, Kathryn Lee, Vijay Korwar, Daniel Hughes, George Hill, Mohammed Hamdan, Hannah Gould Brown, James Glasbey, Caroline Fryer, Simon Davey, David Cotton, Benjamin Byrne, Oliver Brown, Katarzyna Bera, Joanne Bennett, Richard Bamfor, Danya Bakhbakhi, Muhammad Atif, Elizabeth Armstrong, Piriyankan Ananthavarathan

## Abstract

**Background:**

Accurate assessment of surgical‐site infection (SSI) is crucial for surveillance and research. Self‐reporting patient measures are needed because current SSI tools are limited for assessing patients after leaving hospital. The Bluebelle Wound Healing Questionnaire (WHQ) was developed for patient or observer completion; this study tested its acceptability, scale structure, reliability and validity in patients with closed primary wounds after abdominal surgery.

**Methods:**

Patients completed the WHQ (self‐assessment) within 30 days after leaving hospital and returned it by post. Healthcare professionals completed the WHQ (observer assessment) by telephone or face‐to‐face. Questionnaire response rates and patient acceptability were assessed. Factor analysis and Cronbach's α examined scale structure and internal consistency. Test–retest and self‐ *versus* observer reliability assessments were performed. Sensitivity and specificity for SSI discrimination against a face‐to‐face reference diagnosis (using Centers for Disease Control and Prevention criteria) were examined.

**Results:**

Some 561 of 792 self‐assessments (70·8 per cent) and 597 of 791 observer assessments (75·5 per cent) were completed, with few missing data or problems reported. Data supported a single‐scale structure with strong internal consistency (α greater than 0·8). Reliability between test–retest and self‐ *versus* observer assessments was good (κ 0·6 or above for the majority of items). Sensitivity and specificity for SSI discrimination was high (area under the receiver operating characteristic (ROC) curve 0·91).

**Conclusion:**

The Bluebelle WHQ is acceptable, reliable and valid with a single‐scale structure for postdischarge patient or observer assessment of SSI in closed primary wounds.

## Abstract 2

Simple and valid

## Introduction

Surgical‐site infection (SSI) is the third most common healthcare‐associated infection in the UK^1^ influencing patient outcomes, quality of life and healthcare resources^2^. Rates of SSI vary considerably, depending on the type of surgery performed (for instance, clean or contaminated) and individual patient risk factors. Many SSIs take time to become apparent, often developing or becoming symptomatic after the patient has left hospital^3^, ^4^. Rate estimates are influenced by methods and timing of data collection, particularly the robustness of postdischarge follow‐up^2^, ^5^, ^6^. Accurate assessment after discharge is therefore key to SSI surveillance and research is needed to minimize this important healthcare issue^7^.

Assessing wounds for SSI after hospital discharge can be done by patient self‐reporting, by asking patients to return for an outpatient appointment, or by conducting home visits. The latter two methods are resource‐intensive^8^. Patient self‐reporting can reduce these burdens, although accurate tools are needed. Existing postdischarge self‐reporting questionnaires for patients^9^, ^10^, ^11^ have methodological weaknesses. They have been adapted from tools intended for completion by a professional, lack patient input in their development, have not been validated for use in a postdischarge setting, and have been criticized because they do not account for symptom severity – an important aspect in SSI diagnosis^12^. The Bluebelle Wound Healing Questionnaire (WHQ) was developed with input from patients and multidisciplinary healthcare professionals to address these limitations. It assesses signs, symptoms and wound care interventions relevant for the diagnosis of SSI in closed primary wounds, specifically after the patient has left hospital^13^. Early work^13^ has demonstrated that the WHQ is comprehensive, easily understood, and can be completed by patients and/or observers (healthcare professionals). The present study examined the acceptability, scale structure, reliability and validity of the WHQ in a large sample of patients undergoing surgery with closed primary abdominal wounds.

## Methods

### The Bluebelle Wound Healing Questionnaire

The WHQ was developed as part of the Bluebelle study^14^, a feasibility study that included a pilot RCT to examine whether an RCT of different wound dressing strategies for reducing SSI was possible^14^, ^15^. Initial development of the WHQ has been reported previously^13^. The WHQ was designed as a single questionnaire for patient and/or observer completion.

The version of the questionnaire undergoing validation in this study consisted of 16 items: eight relating to signs and symptoms of SSI, and eight relating to wound care interventions. Two of these items included additional components, collecting more detail on signs and symptoms, if applicable. Early versions of the questionnaire also included questions on resource use (for the wider Bluebelle feasibility study) that were not relevant to the diagnosis of SSI, and therefore are not included in these analyses.

Response categories for sign and symptom items were: ‘not at all’ (score 0), ‘a little’ (1), ‘quite a bit’ (2) and ‘a lot’ (3). Response categories for wound care intervention items were: ‘yes’ (score 1), ‘no’ (0) and ‘don't know’. Higher scores, therefore, indicated more problems.

### Study design

Two data sets from the Bluebelle study were used in this analysis: data from a cohort recruited specifically to validate this new measure; and data from the pilot RCT. Research ethical approval was granted from the National Health Service (NHS) Health Research Authority National Research Ethics Service (NRES) Committee London – Camden and Kings Cross (reference 14/LO/0640) and the South West – Frenchay Research Ethics Committee (reference 15/SW/0008).

Eligible participants were aged over 16 years, undergoing elective or unplanned abdominal general surgery or caesarean section. Participants who lacked capacity, ability to read or understand English, and prisoners were excluded. Further inclusion and exclusion criteria were relevant to the wider requirements of the Bluebelle study and have been reported previously^14^. Studies ran between August 2015 and January 2016, and between March 2016 and November 2016 (cohort study and pilot RCT respectively) from four UK NHS hospital trusts. Participants were recruited by research nurses, surgical trainees or other trained members of the study team on hospital wards before or after surgery. Potential participants were given an information leaflet, and were provided with sufficient time to consider involvement and discuss the study before being approached again to take part. All participants were asked to give written informed consent.

### Data collection

**Figure 1:**
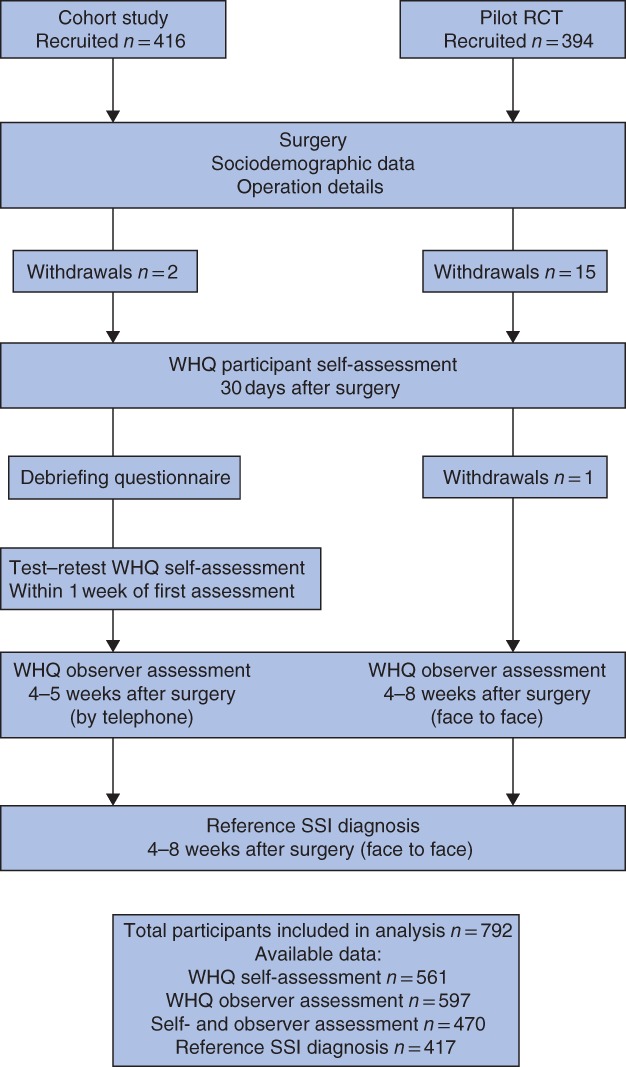
Participants and data contributing to validation of the Wound Healing Questionnaire. WHQ, Wound Healing Questionnaire; SSI, surgical‐site infection

*Fig*. 1 illustrates the study design and data collection.

### Wound Healing Questionnaire self‐assessment

The WHQ was distributed by post for participants to complete and return (by stamped addressed envelope, included) 30 days after surgery. Instructions were to complete the WHQ in relation to events since hospital discharge. A subset of 50 cohort participants (sampled during 1 month of the study) were posted an additional WHQ within 1 week of completing the first WHQ (for test–retest assessment). In a series of debriefing questions included with the WHQ, data were collected from the cohort participants on the time needed for WHQ completion, whether help was required, and whether items were confusing or difficult to answer. Reminders for non‐responders were sent only to participants of the pilot RCT.

### Wound Healing Questionnaire observer assessment

In the cohort study, the WHQ was completed by a clinical member of the study team via a telephone call with participants 4–5 weeks after surgery. In the pilot RCT, the WHQ was completed by a clinical member of the study team during the participant's face‐to‐face follow‐up appointment between 4 and 8 weeks after surgery.

Reference diagnoses of whether SSI had occurred since the time of surgery were made in face‐to‐face study follow‐up appointments between 4 and 8 weeks after the operation using Centers for Disease Control and Prevention (CDC) criteria and classification of no SSI, superficial, deep or organ/space^16^. Diagnoses were made by an independent member of the study team, blinded to the WHQ self‐ and observer assessment, using any available sources of information from the participant and hospital records. All pilot RCT participants and a convenience sample of cohort participants (sampled by availability due to limited study resources) underwent a face‐to‐face reference wound assessment.

### Analyses

All 16 items were included in the initial analysis. Missing responses to the items with multiple components (collecting more detail on signs and symptoms, if applicable) were imputed with values of zero if no response was expected (for example, if the sign or symptom had not occurred). Summation of item scores was performed as suggested by the data. Reference SSI diagnoses were dichotomized to create a binary variable with 0 = no SSI and 1 = SSI of any type (combining CDC classifications of superficial, deep and organ/space SSI due to low numbers of reported deep and organ/space SSI).

#### *Acceptability*

Acceptability of the WHQ was explored in three ways: first, by examining response rates (the proportion of completed WHQ self‐ and observer assessments); second, by exploring missing responses to individual items (indicating possible issues such as not understanding the item); and third, by examining answers to the debriefing questions.

#### *Scale structure*

Exploratory factor analyses examined the underlying structure and constructs of the questionnaire. Analyses were conducted separately for self‐ and observer data. First, all iterations of item pairs were explored using Pearson's correlation coefficients. Pairs with very high correlations (*r* = 0·9 or above) were examined for similarity and considered for redundancy and exclusion before conducting factor analyses^17^. Next, three separate factor analysis models were run, specifying the maximum number of factors to be retained as one, two and three factors (maximum‐likelihood method of estimation). Models were initially explored with data from the cohort study, and the best‐fitting model was applied to data from the pilot RCT as a method of independent validation of the scale structure. The best‐fitting model was applied finally to the combined cohort and pilot RCT data. A sensitivity factor analysis was performed using a polychoric matrix because of the ordinal, categorical nature of the WHQ data^18^. Multitrait scaling analyses were also applied as a comparative statistical approach^19^.

Internal consistency (internal reliability) of the scales identified from the factor analyses was examined using Cronbach's α coefficient^17^. Values greater than 0·7 were considered to have good internal consistency^17^.

#### *Reliability*

Test–retest reliability^20^ was assessed by comparing self‐assessment responses to the WHQ completed twice over a period of anticipated stable health. Stable health was assumed if responders reported that they had not been back into hospital for treatment with a problem with the wound (item 11) in the retest assessment. Cross‐tabulations of responses and weighted κ statistics were calculated. Equal weights between response categories for ordinal items (items 1–8) were assumed, with weighted values of 0, 0·333, 0·667 and 1 between categories. κ values below 0·4 were considered to indicate poor agreement. Values between 0·4 and 0·75 were considered to indicate fair to good agreement^17^.

Inter‐rater reliability (agreement between self‐ and observer assessments, where data from both assessments were available) was explored, to examine the reliability of the self‐assessment for collecting outcome data in a future large‐scale trial. Cross‐tabulations of item responses and weighted κ statistics were calculated as described above. Percentages of agreement and discordance were examined.

#### *Validity*

Criterion validity was examined against the reference SSI diagnosis to demonstrate how well the WHQ performed in discriminating between individuals with and those without SSI. Cross‐tabulations of the reference CDC diagnosis (‘no SSI’ or ‘SSI of any type’) and a binary variable of the self‐assessment WHQ total score (created by a cut‐off score; for instance, a WHQ total score of less than or equal to *x*) were compared. Sensitivity and 1 − specificity values of the WHQ for different cut‐off scores were used to plot a receiver operating characteristic (ROC) curve, representing the trade‐off between sensitivity and specificity^21^.

The overall ability of the WHQ to discriminate between individuals with and those without SSI was measured by the area under the ROC curve (AUC) and 95 per cent confidence intervals. An AUC value approaching 1·0 was interpreted to indicate good discrimination with high sensitivity and specificity, whereas a value of 0·5 was interpreted as the measure not being able to discriminate at all^21^.

Analyses were performed using STATA^®^ statistical software version 14 (StataCorp, College Station, Texas, USA).

### Modifications for the final questionnaire

Findings from the above were used to inform modifications to the final version of the WHQ, considering rates of missing data for individual items, answers to the debriefing questions and overlap between items (if correlations of *r* greater than 0·9 were observed).

## Results

Data for 792 participants were examined (*Fig*. 1). *Table* 1 presents participant sociodemographic, clinical and operative details. Median times from surgery to participant self‐ and observer WHQ assessments were 29 (i.q.r. 24–33) and 37 (32–48) days respectively.

**Table 1:** Baseline sociodemographic, clinical and operative details of the study sample

	No. of patients* (*n* = 792)
Age (years)†	53·2(17·5)
No. of men	364 (46·0)
Duration of surgery (h)	
< 1	213 (28·3)
1–2	182 (24·2)
2–3	139 (18·5)
> 3	218 (29·0)
Missing	40
Type of operation	
Caesarean section	95 (12·2)
Oesophagogastric resection/gastrectomy	17 (2·2)
Pancreatobiliary resection	38 (4·9)
Antireflux surgery	12 (1·5)
Bariatric surgery	6 (0·8)
Cholecystectomy	102 (13·1)
Colectomy/hemicolectomy	95 (12·2)
Hartmann procedure/reversal	21 (2·7)
Rectal/anterior resection	72 (9·2)
Stoma formation alone	11 (1·4)
Stoma closure/reversal alone	19 (2·4)
Small bowel resection	38 (4·9)
Groin hernia repair	61 (7·8)
Abdominal wall hernia repair	37 (4·7)
Appendicectomy	57 (7·3)
Diagnostic laparoscopy/laparotomy	31 (4·0)
Adhesiolysis	12 (1·5)
Other	56 (7·2)
Missing	12
Type of surgery	
Elective	606 (81·3)
Unplanned	139 (18·7)
Missing	47
Risk factor	
Smoker	
Current	114 (14·7)
Ex‐smoker < 1 month	236 (30·4)
No	426 (54·9)
Missing	16
Diabetes, any type (*n* = 775)	60 (7·7)
ASA grade	
I	232 (31·7)
II	373 (51·0)
III	118 (16·1)
IV	8 (1·1)
Missing	61
BMI (kg/m^2^)† (*n* = 762)	28·0(6·1)

* With percentages as proportions of available data (excluding missing values) in parentheses unless indicated otherwise;

† values are mean(s.d.).

### Acceptability

#### *Response rates*

Self‐ and observer WHQ assessments were completed for 561 of 792 (70·8 per cent) and 597 of 791 (75·5 per cent) participants respectively, with 470 of 791 (59·4 per cent) of these participants having both sets of data completed. In total, 104 of 792 participants (13·1 per cent) did not have any WHQ self‐ or observer assessments available (complete non‐responders).

#### *Missing responses to items*

Less than 3 per cent of responses were missing for most items (10 of 16) in the self‐assessments and no items had more than 4 per cent of responses missing (*Table* S1, supporting information). For observer assessments, nearly all items (15 of 16) had less than 2 per cent of responses missing. Missing responses to the additional components of the two items for which further information on signs and symptoms was intended to be collected (if applicable) were, however, high, with up to 43 per cent of self‐assessments missing a response when one would have been expected. Missing responses to these additional components were lower in the observer assessments, although levels were still notable and ranged between 8 and 17 per cent (*Table* S1, supporting information).

#### *Responses to debriefing questions*

Most participants (276 of 302, 91·4 per cent) reported that the questionnaire took fewer than 10 min to complete. Less than 6 per cent reported needing help or finding items difficult or confusing to answer.

### Scale structure

A high correlation (*r* = 0·95) was observed between item 4 (‘Have the edges of any part of the wound separated/gaped open of their own accord? (spontaneous dehiscence)’) and its additional component collecting further information: 4a (‘Did the skin separate?’). Study team agreement of similarity in the underlying concept of these questions deemed item 4a to be redundant, and it was therefore excluded from factor analyses.

Factor analyses of the cohort and pilot RCT data separately supported a single‐scale structure. Results from the combined data set are shown in *Table* 2. Item factor loadings ranged between 0·32 and 0·87 in data from participant self‐assessments, and between 0·33 and 0·85 in data from observer assessments (with the exception of one item with a factor loading of 0·03). Examination of eigenvalues and factor loadings provided little evidence to suggest a better fit for a two‐ or three‐factor model. Sensitivity analyses using a polychoric correlation matrix supported findings for a single‐scale model. A comparative multitrait scaling analysis approach also demonstrated strong association of items to a single scale.

**Table 2:** Factor analysis: item factor loadings for a single‐scale structure using combined cohort and pilot RCT data

		Self‐assessment (*n* = 362)	Observer assessment (*n* = 501)
Eigenvalue		5·26	5·08
Item			
1	Was there redness spreading away from the wound? (erythema/cellulitis)	0·45	0·66
2	Was the area around the wound warmer than the surrounding skin?	0·32	0·56
3	Was any part of the wound leaking fluid?	0·87	0·85
3a	Was it clear fluid? (serous exudate)	0·57	0·45
3b	Was it blood‐stained fluid? (haemoserous exudate)	0·72	0·58
3c	Was it thick and yellow/green fluid (pus/purulent exudate)	0·57	0·64
4	Have the edges of any part of the wound separated/gaped open of their own accord? (spontaneous dehiscence)	0·66	0·63
4a	Did the deeper tissue separate?	0·59	0·43
5	Has the area around the wound become swollen?	0·32	0·36
6	Has the wound been smelly?	0·49	0·43
7	Has the wound been painful to touch?	0·36	0·37
8	Have you had, or felt like you have had, a raised temperature or fever? (fever > 38 °C)	0·39	0·39
9	Have you sought advice because of a problem with your wound, other than at a routine planned follow‐up appointment?	0·61	0·59
10	Has anything been put on the skin to cover the wound? (dressing)	0·42	0·54
11	Have you been back into hospital for treatment of a problem with your wound?	0·45	0·35
12	Have you been given antibiotics for a problem with your wound?	0·65	0·67
13	Have the edges of your wound been deliberately separated by a doctor or nurse?	0·41	0·40
14	Has your wound been scraped or cut to remove any unwanted tissue? (debridement of wound)	0·34	0·03
15	Has your wound been drained? (drainage of pus/abscess)	0·38	0·33
16*	Have you had an operation under general anaesthetic for treatment of a problem with your wound?	–	–

* This item was dropped from the model because of collinearity.

Data suggested it was sensible to calculate a WHQ total score by summing the raw scores for each item without any weightings.

Cronbach's α for a single scale was high, with coefficients of 0·86 in participant data and 0·88 in observer data.

### Reliability

#### *Test–retest reliability*

A total of 44 of 50 participants (88 per cent) included in the test–retest sample (who all reported stable health) completed and returned a second WHQ. The median time between test–retest assessments was 5 (i.q.r. 4–7) days. Agreement in responses for test–retest assessments was high, with levels of observed agreement greater than 86·2 per cent for all items (*Table* S2, supporting information). Where it was possible to calculate a reliable κ statistic, the majority of values were greater than 0·59.

#### *Inter‐rater reliability*

**Figure 2:**
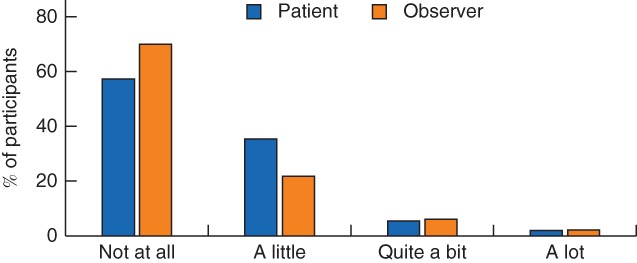
Comparison of responses in self‐ and observer assessments. Example shows the first item in the Wound Healing Questionnaire: ‘Was there redness spreading away from the wound? (erythema/cellulitis)’

Self‐ and observer assessments were available for 59·4 per cent of participants, with a median of 8 (i.q.r. 2–16) days between assessments. Agreement was generally high (observed agreement for any item greater than 84·3 per cent), although participants showed a trend to report levels of signs and symptoms to be slightly more severe than observers; an example from one item is shown in *Fig*. 2 (for data from all items, see *Fig*. S1, supporting information). Where it was possible to calculate a reliable κ statistic, values were between 0·40 and 0·74 for the majority of items (*Table* S3, supporting information). Some minor discrepancy was shown between participant and observer responses to wound‐care intervention items, and whether these interventions had occurred.

### Validity

**Figure 3:**
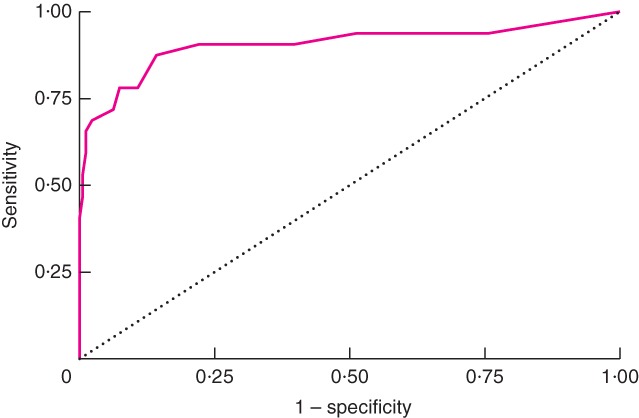
Receiver operating characteristic (ROC) curve for Wound Healing Questionnaire self‐assessment total score for discriminating surgical‐site infection compared with reference diagnosis. Area under ROC curve = 0·91

Reference SSI diagnoses (face‐to‐face, using CDC criteria) were available for 417 of 791 participants (52·7 per cent). Sensitivity and specificity values of the WHQ self‐assessment for discriminating between SSI and no SSI were high, with an area under the ROC curve of 0·91 (95 per cent c.i. 0·83 to 0·98) (*Fig*. 3). Cross‐tabulation of the self‐assessment WHQ total score (excluding item 4a) with the reference SSI diagnosis is provided in *Table* S4 (supporting information). Sensitivity and specificity values for selected WHQ cut‐off scores are shown in *Table* S5 (supporting information). From the present data set, a cut‐off score of 6–8 appeared to be a reasonable threshold for suggesting no SSI/SSI compared with the reference diagnosis, with relatively few misclassifications.

### Modifications for the final questionnaire

Evidence supported the need for minor revisions to the WHQ format to improve its efficiency and minimize missing data. Item 3 and its additional components collecting more information (3a–c) were restructured into three stand‐alone items. Item 4a was removed. Items were renumbered to accommodate these changes. The response option of ‘don't know’ was removed. Questions collecting resource use purely for the purposes of the economic analysis of the Bluebelle pilot RCT were no longer included. The final WHQ items, after these revisions, are shown in *Table* 3.

**Table 3:** Revised Wound Healing Questionnaire items after analysis

Item		Response categories
1	Was there redness spreading away from the wound? (erythema/cellulitis)	Not at all / A little / Quite a bit / A lot
2	Was the area around the wound warmer than the surrounding skin?	Not at all / A little / Quite a bit / A lot
3	Has any part of the wound leaked clear fluid? (serous exudate)	Not at all / A little / Quite a bit / A lot
4	Has any part of the wound leaked blood‐stained fluid? (haemoserous exudate)	Not at all / A little / Quite a bit / A lot
5	Has any part of the wound leaked thick and yellow/green fluid (pus/purulent exudate)	Not at all / A little / Quite a bit / A lot
6i	Have the edges of any part of the wound separated/gaped open of their own accord? (spontaneous dehiscence)	Not at all / A little / Quite a bit / A lot
6ii	Did the deeper tissue separate?	Not at all / A little / Quite a bit / A lot
7	Has the area around the wound become swollen?	Not at all / A little / Quite a bit / A lot
8	Has the wound been smelly?	Not at all / A little / Quite a bit / A lot
9	Has the wound been painful to touch?	Not at all / A little / Quite a bit / A lot
10	Have you had, or felt like you have had, a raised temperature or fever? (fever > 38 °C)	Not at all / A little / Quite a bit / A lot
11	Have you sought advice because of a problem with your wound, other than at a routine planned follow‐up appointment?	Yes / No
12	Has anything been put on the skin to cover the wound? (dressing)	Yes / No
13	Have you been back into hospital for treatment of a problem with your wound?	Yes / No
14	Have you been given antibiotics for a problem with your wound?	Yes / No
15	Have the edges of your wound been deliberately separated by a doctor or nurse?	Yes / No
16	Has your wound been scraped or cut to remove any unwanted tissue? (debridement of wound)	Yes / No
17	Has your wound been drained? (drainage of pus/abscess)	Yes / No
18	Have you had an operation under general anaesthetic for treatment of a problem with your wound?	Yes / No

## Discussion

This study examined the acceptability, scale structure, reliability and validity of the WHQ for use as a patient‐ or observer‐completed tool for the assessment of SSI in closed primary surgical wounds after abdominal surgery. The WHQ was found to be acceptable to patients and demonstrated good response rates, with low levels of missing data. Analyses supported a single‐scale structure to assess SSI that made clinical and practical sense. Test–retest reliability was high, and agreement between participants and observers was good. The WHQ demonstrated high sensitivity and specificity for SSI discrimination compared with a face‐to‐face reference CDC diagnosis. It is therefore suggested that the WHQ is an acceptable, reliable and valid patient‐reported or observer‐completed questionnaire for assessing SSI in closed primary surgical wounds.

Existing self‐reported questionnaires for patients have been adapted mostly from the CDC criteria and ASEPSIS tools^11^, ^16^, ^22^. They are limited because of the lack of user involvement in development. Criticisms include that they are complicated and difficult to complete^11^, ^23^. These self‐reporting measures are also limited in their design, such as asking for yes/no responses to questions without the option to report the amount or severity of the sign/symptom. This is important when assessing a wound, as demonstrated, for example, in a recent study^12^ that found the amount of exudate was more strongly associated with SSI than with the type of exudate. Existing patient measures, however, do not provide an opportunity for the amount of exudate to be captured. The same study also highlighted that bright red skin was observed in patients who had SSI, but also in patients who did not, providing another example where capturing the amount or severity of a sign/symptom rather than just its presence or absence is important. The WHQ has addressed these limitations by involving a multidisciplinary team (including patients, surgeons, nurses, microbiologists and health service researchers) in its development and by using a combination of qualitative and quantitative methods; it also underwent rigorous pretesting during development to ensure face and content validity^13^. The result is a reliable, valid, comprehensive and uncomplicated questionnaire that includes an ordinal response scale to capture symptom severity.

The study has some limitations. First, a true standard for the diagnosis of SSI without subjective perceptions or opinions is lacking, with the result that tests for criterion validity are limited. The CDC classification of SSI diagnosis was chosen as the best available reference standard for comparing the WHQ as it is the most commonly used and widely regarded tool available. Second, reports of the more major wound care interventions (such as debridement and drainage) were rare in this data set; this may have an impact, for example, on factor analyses. In addition, some discrepancy was observed between participant and observer reports of these major interventions, suggesting possible low fidelity of participant responses. Although these more major interventions were rare in this data set and the number of discordant reports between self‐ and observer assessments were few, this discrepancy may be important to consider and warrants further investigation, as it may have implications for studies relying solely on patient self‐assessment for collecting outcome data. Missing data in responses to the additional component parts of items collecting further information on signs and symptoms (if applicable) were relatively high, although this may be explained by the layout of the questionnaire; modifications in the revised version aim to address this. Although the wide range of abdominal operations is a strength of this study, it is recognized that the proportion of participants undergoing caesarean section (12·2 per cent) is likely to have affected the representative age of the rest of the sample presenting for general abdominal surgery and may have affected the findings. Finally, other limitations of this work relate to its testing and use after abdominal surgery alone, and for wounds healing by primary intention.

Further use and validation of the final version of the WHQ in other types of wound and surgical specialty is underway. Cut‐off scores for SSI diagnosis will be explored. In addition, members of the research group are exploring the feasibility of collecting digital images of the wound taken by patients as a tool to use in conjunction with the WHQ for improving remote and blinded SSI assessment. Advances in digital technology, including the use of smart phones and other tablet devices with cameras, mean that obtaining data from patients after discharge is becoming increasing possible^24^, ^25^. These moves towards using digital technologies to obtain patient‐reported data, including images of wounds, have great potential for improving SSI assessment and ultimately patient care.

## Collaborators

The study group consists of the following: Rhiannon Macefield (co‐led WHQ development and validation; wrote first draft); Jane Blazeby (chief investigator, responsible for concept and design); Barnaby Reeves (study co‐investigator, responsible for pilot RCT design and protocol, and WHQ validation design and analysis); Sara Brookes and Kerry Avery (advised on WHQ validation analysis); Chris Rogers (study co‐investigator, responsible for overall analysis; advised on WHQ validation analysis). All of the above commented on the final draft of the manuscript.

The following authors were Bluebelle study co‐investigators, with further contributions indicated: Mark Woodward (paediatrics); Nicky Welton (value for information analysis); Leila Rooshenas and Jonathan Mathers (qualitative work); Andrew Torrance; Anne Pullyblank; Robert Longman; Richard Lovegrove; Tim Draycott (study delivery); Thomas Pinkney (study delivery, contributed to WHQ development); Rachael Gooberman‐Hill (patient and public involvement); Jenny Donovan (qualitative research); Joanna Coast (health economic analysis); Melanie Calvert (WHQ and other outcome measure development); Natalie Blencowe (survey of wound dressings, contributed to WHQ and other outcome measure development); Lazaros Andronis (health economic analysis).

Other Bluebelle Study Group members: Dimitrios Siassakos (study implementation); Caroline Pope, Madeleine Clout, Kate Ashton and Lucy Ellis (study set up and management); Christel McMullan (qualitative work); Rosie Harris (pilot RCT statistical analysis); Daisy Elliott (development of other study outcome measures); Jo Dumville (Cochrane update review of wound dressings). The following members (surgical trainee collaboratives) all contributed to patient recruitment and study delivery in local hospitals: Benjamin Waterhouse, Sean Strong, William Seligman, Lloyd Rickard, Samir Pathak, Anwar Owais, Jamie O'Callaghan, Stephen O'Brien, Dmitri Nepogodiev, Khaldoun Nadi, Charlotte Murkin, Tonia Munder, Tom Milne (also contributed to WHQ development), David Messenger, Matthew Mason, Morwena Marshall, Jessica Lloyd, Jeffrey Lim, Kathryn Lee, Vijay Korwar, Daniel Hughes, George Hill, Mohammed Hamdan, Hannah Gould Brown, James Glasbey, Caroline Fryer, Simon Davey, David Cotton, Benjamin Byrne, Oliver Brown, Katarzyna Bera, Joanne Bennett, Richard Bamford, Danya Bakhbakhi, Muhammad Atif, Elizabeth Armstrong, Piriyankan Ananthavarathan.

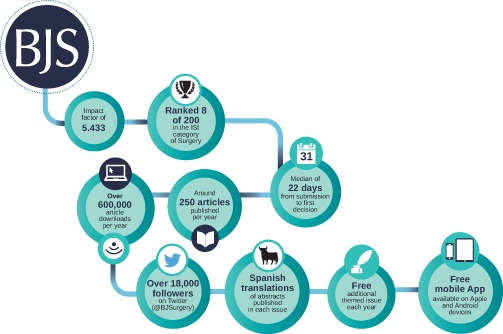

## Supporting information


**Fig. S1** Comparison of responses to items in self‐ and observer assessments, for participants with data from both assessments (*n* = 470)
**Table S1** Distribution of responses and missing data for each item in participant self‐assessments (*n* = 561) and observer assessments (*n* = 597)
**Table S2** Test–retest reliability in participant self‐assessments (*n* = 44)
**Table S3** Self‐ and observer agreement for participants with data from both assessments (*n* = 470)
**Table S4** Cross‐tabulation of self‐assessment WHQ total score and face‐to‐face reference SSI diagnoses
**Table S5** Sensitivity and specificity of selected self‐assessment WHQ total score cut‐off thresholds compared to the reference SSI diagnosis
