# Supplementary material for: Validation of the Bluebelle Wound Healing Questionnaire for assessment of surgical‐site infection in closed primary wounds after hospital discharge
Source: Br J Surg. 2018 Dec 17;106(3):226–35. doi: 10.1002/bjs.11008 (PMC6457211; doi:10.1002/bjs.11008)
Supplement: Supplementary file 1 — Fig. S1 Comparison of responses to items in self‐ and observer assessments, for participants with data from both assessments (n = 470) Table S1 Distribution of responses and missing data for each item in participant self‐assessments (n = 561) and observer assessments (n = 597) Table S2 Test–retest reliability in participant self‐assessments (n = 44) Table S3 Self‐ and observer agreement for participants with data from both assessments (n = 470) Table S4 Cross‐tabulation of self‐assessment WHQ total score and face‐to‐face reference SSI diagnoses Table S5 Sensitivity and specificity of selected self‐assessment WHQ total score cut‐off thresholds compared to the reference SSI diagnosis [file BJS-106-226-s001.docx]

**BJS_11008**

**Validation of the Bluebelle Wound Healing Questionnaire for assessment of surgical-site infection in closed primary wounds after hospital discharge**

**Bluebelle Study Group**

**Fig. S1** Comparison of responses to items in self- and observer assessments, for participants with data from both assessments (*n* = 470)

**Table S1** Distribution of responses and missing data for each item in participant self-assessments (*n* = 561) and observer assessments (*n* = 597)

|  | | **Missing response*** | | **Distribution of responses^**^** | | | | | | | |
| --- | --- | --- | --- | --- | --- | --- | --- | --- | --- | --- | --- |
|  | |  |  | **Not at all** | | **A little** | | **Quite a bit** | | **A lot** | |
| **Item** | | **n** | **(%)** | **n** | **(%)** | **n** | **(%)** | **n** | **(%)** | **n** | **(%)** |
| 1 | Was there redness spreading away from the wound? (erythema/cellulitis) |  |  |  |  |  |  |  |  |  |  |
|  | Participant self-assessments | 8 | (1.4) | 314 | (56.8) | 192 | (34.7) | 36 | (6.5) | 11 | (2.0) |
|  | Observer assessments | 1 | (0.2) | 416 | (69.8) | 130 | (21.8) | 33 | (5.5) | 17 | (2.9) |
| 2 | Was the area around the wound warmer than the surrounding skin? |  |  |  |  |  |  |  |  |  |  |
|  | Participant self-assessments | 19 | (3.4) | 312 | (57.6) | 189 | (34.9) | 32 | (5.9) | 9 | (1.7) |
|  | Observer assessments | 1 | (0.2) | 444 | (74.5) | 108 | (18.1) | 34 | (5.7) | 10 | (1.7) |
| 3 | Was any part of the wound leaking fluid? |  |  |  |  |  |  |  |  |  |  |
|  | Participant self-assessments | 12 | (2.1) | 351 | (63.9) | 125 | (22.8) | 45 | (8.2) | 28 | (5.1) |
|  | Observer assessments | 7 | (1.2) | 419 | (71.0) | 116 | (19.7) | 26 | (4.4) | 29 | (4.9) |
| 3a | Was it clear fluid? (serous exudate) |  |  |  |  |  |  |  |  |  |  |
|  | Participant self-assessments | 86 | (43.4) | 48 | (40.3) | 51 | (42.9) | 17 | (14.3) | 3 | (2.5) |
|  | Observer assessments | 23 | (13.5) | 14 | (51.6) | 58 | (37.4) | 10 | (6.5) | 7 | (4.5) |
| 3b | Was it blood-stained fluid? (haemoserous exudate) |  |  |  |  |  |  |  |  |  |  |
|  | Participant self-assessments | 47 | (23.7) | 45 | (28.5) | 77 | (48.7) | 24 | (15.2) | 12 | (7.6) |
|  | Observer assessments | 14 | (8.2) | 77 | (46.7) | 56 | (33.9) | 18 | (10.9) | 14 | (8.5) |
| 3c | Was it thick and yellow/green fluid? (pus/purulent exudate) |  |  |  |  |  |  |  |  |  |  |
|  | Participant self-assessments | 81 | (41.0) | 71 | (57.3) | 27 | (21.8) | 18 | (14.5) | 8 | (6.5) |
|  | Observer assessments | 23 | (13.5) | 96 | (61.9) | 30 | (19.4) | 13 | (8.4) | 16 | (10.3) |
| 4 | Have the edges of any part of the wound separated/gaped open on their own accord? (spontaneous dehiscence) |  |  |  |  |  |  |  |  |  |  |
|  | Participant self-assessments | 1.4 | (3.4) | 423 | (78.0) | 93 | (17.2) | 17 | (3.1) | 9 | (1.7) |
|  | Observer assessments | 12 | (2.0) | 489 | (83.6) | 76 | (13.0) | 11 | (1.9) | 9 | (1.5) |
| 4a | Did the skin separate? |  |  |  |  |  |  |  |  |  |  |
|  | Participant self-assessments | 6 | (5.0) | 41 | (28.5) | 78 | (52.4) | 20 | (13.4) | 10 | (6.7) |
|  | Observer assessments | 6 | (6.3) | 17 | (15.6) | 71 | (65.1) | 11 | (10.1) | 10 | (9.2) |
| 4b | Did the deeper tissue separate? |  |  |  |  |  |  |  |  |  |  |
|  | Participant self-assessments | 27 | (22.7) | 93 | (75.6) | 15 | (12.2) | 11 | (8.9) | 4 | (3.3) |
|  | Observer assessments | 16 | (16.7) | 77 | (79.4) | 10 | (10.3) | 4 | (4.1) | 6 | (6.2) |
| 5 | Has the area around the wound become swollen? |  |  |  |  |  |  |  |  |  |  |
|  | Participant self-assessments | 14 | (2.5) | 345 | (63.1) | 160 | (29.3) | 35 | (6.4) | 7 | (1.3) |
|  | Observer assessments | 1 | (0.2) | 481 | (80.7) | 96 | (16.1) | 12 | (2.0) | 7 | (1.2) |
| 6 | Has the wound been smelly? |  |  |  |  |  |  |  |  |  |  |
|  | Participant self-assessments | 22 | (3.9) | 488 | (90.5) | 36 | (6.7) | 9 | (1.7) | 6 | (1.1) |
|  | Observer assessments | 1 | (0.2) | 547 | (91.8) | 31 | (5.2) | 13 | (2.2) | 5 | (0.8) |
| 7 | Has the wound been painful to touch? |  |  |  |  |  |  |  |  |  |  |
|  | Participant self-assessments | 13 | (2.3) | 207 | (37.8) | 274 | (50.0) | 50 | (9.1) | 17 | (3.1) |
|  | Observer assessments | 0 | (0) | 351 | (58.8) | 180 | (30.2) | 51 | (8.5) | 15 | (2.5) |
| 8 | Have you had, or felt like you have had, a raised temperature or fever? (fever >38^o^C) |  |  |  |  |  |  |  |  |  |  |
|  | Participant self-assessments | 20 | (3.6) | 462 | (85.4) | 57 | (10.5) | 11 | (2.0) | 11 | (2.0) |
|  | Observer assessments | 1 | (0.2) | 524 | (87.9) | 37 | (6.2) | 15 | (2.5) | 20 | (3.4) |
|  |  |  |  |  |  |  |  |  |  |  |  |
|  |  | **Missing** | | **No** | | **Yes** | |  |  |  |  |
|  |  | **n** | **(%)** | **n** | **(%)** | **n** | **(%)** |  |  |  |  |
| 9 | Have you sought advice because of a problem with your wound, other than at a routine planned follow-up appointment? |  |  |  |  |  |  |  |  |  |  |
|  | Participant self-assessments | 4 | (0.7) | 396 | (71.1) | 161 | (28.9) |  |  |  |  |
|  | Observer assessments | 8 | (1.3) | 442 | (75.0) | 147 | (25.0) |  |  |  |  |
| 10 | Has anything been put on the skin to cover the wound? (dressing) |  |  |  |  |  |  |  |  |  |  |
|  | Participant self-assessments | 6 | (1.1) | 333 | (60.0) | 222 | (40.0) |  |  |  |  |
|  | Observer assessments | 4 | (0.7) | 396 | (66.8) | 197 | (33.2) |  |  |  |  |
| 11 | Have you been back into hospital for treatment with a problem with your wound? |  |  |  |  |  |  |  |  |  |  |
|  | Participant self-assessments | 17 | (3.0) | 514 | (94.5) | 30 | (5.5) |  |  |  |  |
|  | Observer assessments | 22 | (3.7) | 548 | (95.3) | 27 | (4.7) |  |  |  |  |
|  |  |  |  |  |  |  |  |  |  |  |  |
|  |  | **Missing** | | **No** | | **Yes** | | **Don’t know** | |  |  |
|  |  | **n** | **(%)** | **n** | **(%)** | **n** | **(%)** | **n** | **(%)** |  |  |
| 12 | Have you been given antibiotics for a problem with your wound? |  |  |  |  |  |  |  |  |  |  |
|  | Participant self-assessments | 9 | (1.6) | 463 | (83.9) | 82 | (14.9) | 7 | (1.3) |  |  |
|  | Observer assessments | 5 | (0.8) | 511 | (86.3) | 81 | (13.7) | 0 | (0) |  |  |
| 13 | Have the edges of your wound been deliberately separated by a doctor or nurse? |  |  |  |  |  |  |  |  |  |  |
|  | Participant self-assessments | 7 | (1.3) | 532 | (96.0) | 16 | (2.9) | 6 | (1.1) |  |  |
|  | Observer assessments | 4 | (0.7) | 572 | (96.5) | 21 | (3.5) | 0 | (0) |  |  |
| 14 | Has your wound been scraped or cut to remove any unwanted tissue? (debridement of wound) |  |  |  |  |  |  |  |  |  |  |
|  | Participant self-assessments | 13 | (2.3) | 539 | (98.4) | 6 | (1.1) | 3 | (0.6) |  |  |
|  | Observer assessments | 3 | (0.5) | 588 | (99.0) | 6 | (1.0) | 0 | (0) |  |  |
| 15 | Has your wound been drained? (drainage of pus/abscess) |  |  |  |  |  |  |  |  |  |  |
|  | Participant self-assessments | 18 | (3.2) | 518 | (95.4) | 21 | (3.9) | 4 | (0.74) |  |  |
|  | Observer assessments | 5 | (0.8) | 580 | (98.0) | 11 | (1.9) | 1 | (0.2) |  |  |
| 16 | Have you had an operation under general anaesthetic for treatment of a problem with your wound? |  |  |  |  |  |  |  |  |  |  |
|  | Participant self-assessments | 15 | (2.7) | 542 | (99.3) | 2 | (0.4) | 2 | (0.4) |  |  |
|  | Observer assessments | 7 | (1.2) | 590 | (100) | 0 | (0) | 0 | (0) |  |  |

*Percentages of missing responses were calculated as the proportion missing in an otherwise completed WHQ (n=561 and n=597, self- and observer assessments respectively). For questions 3a-c and 4a-b collecting additional information, percentages were calculated as the proportion missing where a response would have been expected (i.e. responses to items 3 and 4 that were anything other than ‘not at all’ (patient self-assessments n=198 and n=119, respectively; observer assessments n=171 and n=96, respectively)

**Percentages were calculated as the proportion of available data (excluding missing responses)

**Table S2** Test–retest reliability in participant self-assessments (*n* = 44)

| **Item** | | **n** | **% observed agreement** | **% expected agreement** | **Weighted Kappa** | **% agreed responses that were ‘Not at all’ or ‘No’** | **Missing** |
| --- | --- | --- | --- | --- | --- | --- | --- |
| 1 | Was there redness spreading away from the wound? (erythema/cellulitis) | 41 | 86.2 | 80.1 | 0.3051 | 39.0 | 1 (first test)  2 (retest) |
| 2 | Was the area around the wound warmer than the surrounding skin? | 38 | 88.6 | 82.5 | 0.3500 | 47.4 | 2 (first test)  5 (retest) |
| 3 | Was any part of the wound leaking fluid? | 43 | 92.3 | 73.8 | 0.7043 | 55.8 | 1 (retest) |
| 3a | Was it clear fluid? (serous exudate) | 4 | 100 | 87.5 | 1.000 | 75.0 | 6 (first test)  9 (retest) |
| 3b | Was it blood-stained fluid? (haemoserous exudate) | 11 | 93.9 | 74.1 | 0.7660 | 14.3 | 2 (first test)  3 (retest) |
| 3c | Was it thick and yellow/green fluid (pus/purulent exudate) | 5 | 86.7 | 54.7 | 0.7059 | 14.3 | 5 (first test)  6 (retest) |
| 4 | Have the edges of any part of the wound separated/gaped open on their own accord? (spontaneous dehiscence) | 43 | 97.7 | 84.4 | 0.8509 | 74.4 | 1 (retest) |
| 4a | Did the skin separate? | 8 | 100 | 87.5 | 1.00 | 75.0 | 1 (first test) |
| 4b | Did the deeper tissue separate? | 6 | 100 | 87.5 | 1.00 | 83.3 | 3 (first test)  3 (retest) |
| 5 | Has the area around the wound become swollen? | 44 | 93.2 | 81.8 | 0.6250 | 59.1 | 0 |
| 6 | Has the wound been smelly? | 43 | 97. 7 | 96.4 | 0.3645 | 90.7 | 1 (first test)  1 (retest) |
| 7 | Has the wound been painful to touch? | 43 | 91.5 | 79.4 | 0.5854 | 37.2 | 1 (retest) |
| 8 | Have you had, or felt like you have had, a raised temperature or fever? (fever >38^o^C) | 43 | 96.1 | 93.7 | 0.3804 | 83.7 | 1 (retest) |
| 9 | Have you sought advice because of a problem with your wound, other than at a routine planned follow-up appointment? | 43 | 86.15 | 56.0 | 0.6830 | 60.5 | 1 (first test) |
| 10 | Has anything been put on the skin to cover the wound? (dressing) | 44 | 97.7 | 50.6 | 0.9540 | 43.2 | 0 |
| 11 | Have you been back into hospital for treatment with a problem with your wound? | 44 | 100 | 83.5 | 1.000 | 90.9 | 0 |
| 12 | Have you been given antibiotics for a problem with you wound? | 43 | 95.4 | 75.9 | 0.8072 | 83.7 | 1 (retest) |
| 13 | Have the edges of your wound been deliberately separated by a doctor or nurse? | 43 | 97.7 | 97.7 | 0.0000 | 95.5 | 1 don’t know (retest) |
| 14 | Has your wound been scraped or cut to remove any unwanted tissue? (debridement of wound) | 39 | 94.9 | 95.0 | -0.0263 | 92.5 | 1 (first test)  3 (retest)  1 don’t know (retest) |
| 15 | Has your wound been drained? (drainage of pus/abscess) | 41 | 100 | 95.2 | 1.000 | 97.6 | 1 (first test)  2 (retest) |
| 16^†^ | Have you had an operation under general anaesthetic for treatment of a problem with your wound? | 40 | 100 | - | - | 100 | 2 (first test)  2 (retest) |

^†^Expected agreement and Kappa statistic not possible to compute as all observations were of the same category (‘No’)

**Table S3** Self- and observer agreement for participants with data from both assessments (*n* = 470)

| **Item** | | **n** | **% observed agreement** | **% expected agreement** | **Weighted Kappa** | **% agreed responses that were ‘Not at all’ or ‘No’** |
| --- | --- | --- | --- | --- | --- | --- |
| 1 | Was there redness spreading away from the wound? (erythema/cellulitis) | 463 | 87.5 | 78.2 | 0.4260 | 50.3 |
| 2 | Was the area around the wound warmer than the surrounding skin? | 454 | 89.1 | 79.8 | 0.4597 | 54.9 |
| 3 | Was any part of the wound leaking fluid? | 458 | 94.0 | 76.5 | 0.7432 | 63.3 |
| 3a | Was it clear fluid? (serous exudate) | 67 | 90.6 | 71.4 | 0.6691 | 29.9 |
| 3b | Was it blood-stained fluid? (haemoserous exudate) | 89 | 86.7 | 68.6 | 0.5784 | 22.5 |
| 3c | Was it thick and yellow/green fluid (pus/purulent exudate) | 71 | 84.7 | 68.5 | 0.5143 | 42.3 |
| 4 | Have the edges of any part of the wound separated/gaped open on their own accord? (spontaneous dehiscence) | 447 | 95.2 | 86.9 | 0.6314 | 76.5 |
| 4a | Did the skin separate? | 56 | 88.6 | 74.6 | 0.5501 | 0 |
| 4b | Did the deeper tissue separate? | 43 | 91.0 | 74.7 | 0.6456 | 62.8 |
| 5 | Has the area around the wound become swollen? | 459 | 88.2 | 82.9 | 0.3092 | 59.0 |
| 6 | Has the wound been smelly? | 454 | 97.4 | 93.7 | 0.5911 | 89.9 |
| 7 | Has the wound been painful to touch? | 462 | 84.3 | 75.0 | 0.3748 | 31.6 |
| 8 | Have you had, or felt like you have had, a raised temperature or fever? (fever >38^o^C) | 454 | 94.9 | 89.4 | 0.5203 | 83.5 |
| 9 | Have you sought advice because of a problem with your wound, other than at a routine planned follow-up appointment? | 461 | 85.9 | 61.8 | 0.6312 | 67.3 |
| 10 | Has anything been put on the skin to cover the wound? (dressing) | 464 | 84.7 | 53.3 | 0.6721 | 55.4 |
| 11 | Have you been back into hospital for treatment with a problem with your wound? | 437 | 95.7 | 91.1 | 0.5138 | 93.1 |
| 12 | Have you been given antibiotics for a problem with you wound? | 454 | 96.3 | 76.9 | 0.8379 | 83.3 |
| 13 | Have the edges of your wound been deliberately separated by a doctor or nurse? | 456 | 96.9 | 93.6 | 0.5175 | 94.1 |
| 14 | Has your wound been scraped or cut to remove any unwanted tissue? (debridement of wound) | 454 | 98.7 | 97.8 | 0.3936 | 97.8 |
| 15 | Has your wound been drained? (drainage of pus/abscess) | 445 | 97.1 | 94.9 | 0.4221 | 95.1 |
| 16 | Have you had an operation under general anaesthetic for treatment of a problem with your wound? | 449 | 99.8 | 99.8 | 0.0000 | 99.3 |

**Table S4** Cross tabulation of self-assessment WHQ total score and face-to-face reference SSI diagnoses, for participants with complete data from both assessments

|  | **Self-assessment WHQ total score** | | | | | | | | | | | | | | | | | | | | | | |
| --- | --- | --- | --- | --- | --- | --- | --- | --- | --- | --- | --- | --- | --- | --- | --- | --- | --- | --- | --- | --- | --- | --- | --- |
|  | 0 | 1 | 2 | 3 | 4 | 5 | 6 | 7 | 8 | 9 | 10 | 11 | 12 | 13 | 15 | 17 | 18 | 19 | 20 | 26 | 28 | 30 | **Total** |
| **Reference diagnosis** |  |  |  |  |  |  |  |  |  |  |  |  |  |  |  |  |  |  |  |  |  |  |  |
| No SSI | 43 | 43 | 20 | 31 | 14 | 6 | 6 | 2 | 7 | 2 | 0 | 1 | 0 | 1 | 0 | 0 | 0 | 0 | 0 | 0 | 0 | 0 | **176** |
| SSI of any type | 2 | 0 | 1 | 0 | 1 | 3 | 0 | 2 | 1 | 1 | 2 | 2 | 2 | 2 | 4 | 3 | 1 | 1 | 1 | 1 | 1 | 1 | **32** |
| Total | 45 | 43 | 21 | 31 | 15 | 9 | 6 | 4 | 8 | 3 | 2 | 3 | 2 | 3 | 4 | 3 | 1 | 1 | 1 | 1 | 1 | 1 | **208** |

**Table S5** Sensitivity and specificity of selected self-assessment WHQ total score cut-off thresholds compared to the reference SSI diagnosis

| **WHQ self-assessment score**  **cut-off threshold** | **Sensitivity (%)** | **Specificity (%)** |
| --- | --- | --- |
| 5 | 87.5 | 85.8 |
| 6 | 78.1 | 89.2 |
| 7 | 78.1 | 92.6 |
| 8 | 71.9 | 93.8 |
| 9 | 68.8 | 97.7 |
| 10 | 65.6 | 98.9 |
